# Alzheimer disease and platelets: how’s that relevant

**DOI:** 10.1186/1742-4933-9-20

**Published:** 2012-09-17

**Authors:** Silvia Catricala, Mauro Torti, Giovanni Ricevuti

**Affiliations:** 1 Department of Molecular Medicine, Haematology Section, Fondazione IRCCS Policlinico San Matteo, Pavia, Italy; 2 Department of Biochemistry, University of Pavia, Via Bassi 21, Pavia, Italy; 3 Department of Internal Medicine and Therapeutics, Section of Geriatrics, University of Pavia, ASP-IDR S.Margherita, Via Emilia 12, Pavia, 27100, Italy

**Keywords:** Alzheimer disease, Platelets, Amyloid precursor protein.

## Abstract

Alzheimer Disease (AD) is the most common neurodegenerative disorder worldwide, and account for 60% to 70% of all cases of progressive cognitive impairment in elderly patients. At the microscopic level distinctive features of AD are neurons and synapses degeneration, together with extensive amounts of senile plaques and neurofibrillars tangles. The degenerative process probably starts 20–30 years before the clinical onset of the disease. Senile plaques are composed of a central core of amyloid β peptide, Aβ, derived from the metabolism of the larger amyloid precursor protein, APP, which is expressed not only in the brain, but even in non neuronal tissues. More than 30 years ago, some studies reported that human platelets express APP and all the enzymatic activities necessary to process this protein through the same pathways described in the brain. Since then a large number of evidence has been accumulated to suggest that platelets may be a good peripheral model to study the metabolism of APP, and the pathophysiology of the onset of AD. In this review, we will summarize the current knowledge on the involvement of platelets in Alzheimer Disease. Although platelets are generally accepted as a suitable model for AD, the current scientific interest on this model is very high, because many concepts still remain debated and controversial. At the same time, however, these still unsolved divergences mirror a difficulty to establish constant parameters to better defined the role of platelets in AD.

## Introduction

### Molecular features and pathogenesis of alzheimer disease

Alzhemeir’s Disease (AD) is a chronic progressive neurodegenerative disorder characterized by a devastating cognitive and memory decline. It is the most common cause of dementia in the elderly, affecting about 26 million people worldwide, and whose prevalence has been calculated to quadruple by 2050 [1-3].

The first neuropathological case of a patient affected by AD was described over 100 years ago, and the presence of senile plaques and neurofibrillary tangles in the brain, two major hallmarks of AD, were described [4].

Senile plaques are characterized by the abnormal accumulation of amyloid β-peptide (Aβ) in the form of β-plated sheet fibrils, in nervous tissues and in blood vessels [5]. Aβ is a 4 KDa hydrophobic molecule included into a much larger membrane glycoprotein, named amyloid precursor protein, APP [6], from which it is released upon limited proteolysis. Aβ can exist as monomer, dimer, oligomer, protofibril, and fibrillar aggregates [7]. The propensity to self-association of Aβ seems to depend on the peptide’s primary sequence. Indeed the Aβ42, which makes up less than 10% of total Aβ, is more prone to aggregate than the more abundant Aβ40 [8].

Neurofibrillar tangles are mainly composed by a cytoskeletal microtubule-associated protein, called tau, that becomes hyperphosphorylated, dissociates from microtubules, and self-aggregates in the cytosol to form paired helical filaments. However, since hyperphosphorylated tau seems to be present also in other neurodegenerative diseases, and since all the currently identified genetic mutations responsible for AD invariably result in increased formation of fibrillogenic Aβ, the amyloid cascade hypothesis is the most widely accepted event for the pathogenesis of AD [9].

Two forms of AD have been described: a sporadic or senile form, and a familial (FAD) or presenil form. The former one develops in 95-98% of cases [10,11], while the familial cases are limited to only 2-5%.

The onset of the sporadic form of AD typically occurs in patients after 65 years of age old, meanwhile the onset of familial form occurs generally before this age. No genes are directly responsible for the onset of the sporadic AD, but an association with polymorphisms of the gene ApoE have been reported [12,13].

In nervous system APP plays major roles in synaptogenesis and synaptic plasticity. It is expressed in human brain, cerebrospinal fluid [14], kidney, spleen, heart and adrenal tissues [15], but it also is expressed in peripheral circulating cells as platelets [16]. App gene contains 19 exons, and at least 10 different mRNA can be generated by alternative splicing. The most common isoforms inlcude APP695, predominantly expressed in neuronal tissues, and the isoforms APP751 and APP770 achieved by the insertion of a serine protease inhibitory domain of the Kunitz type family [17], which are abundantly expressed in non-neuronal cells [18-20].

APP is a type I transmembrane glycoprotein with a large extracellular N-terminal domain, and a short cytoplasmatic C-terminal domain [21]. APP proteolitic processing is complex and results from the action of two alternative pathways that involve either α-secretase (non-amyloidogenic pathway) or β-secretase (amyloidogenic pathway), besides the γ-secretase complex [22,23]. Only the amyloidogenic pathway generates and releases Aβ peptide, composed by 40–42 aminoacidic residues [24], while in the non-amyloidogenic pathway sAPPα which may have neuroprotective effects, is produced [25].

### Platelets and alzheimer disease

**Figure 1:**
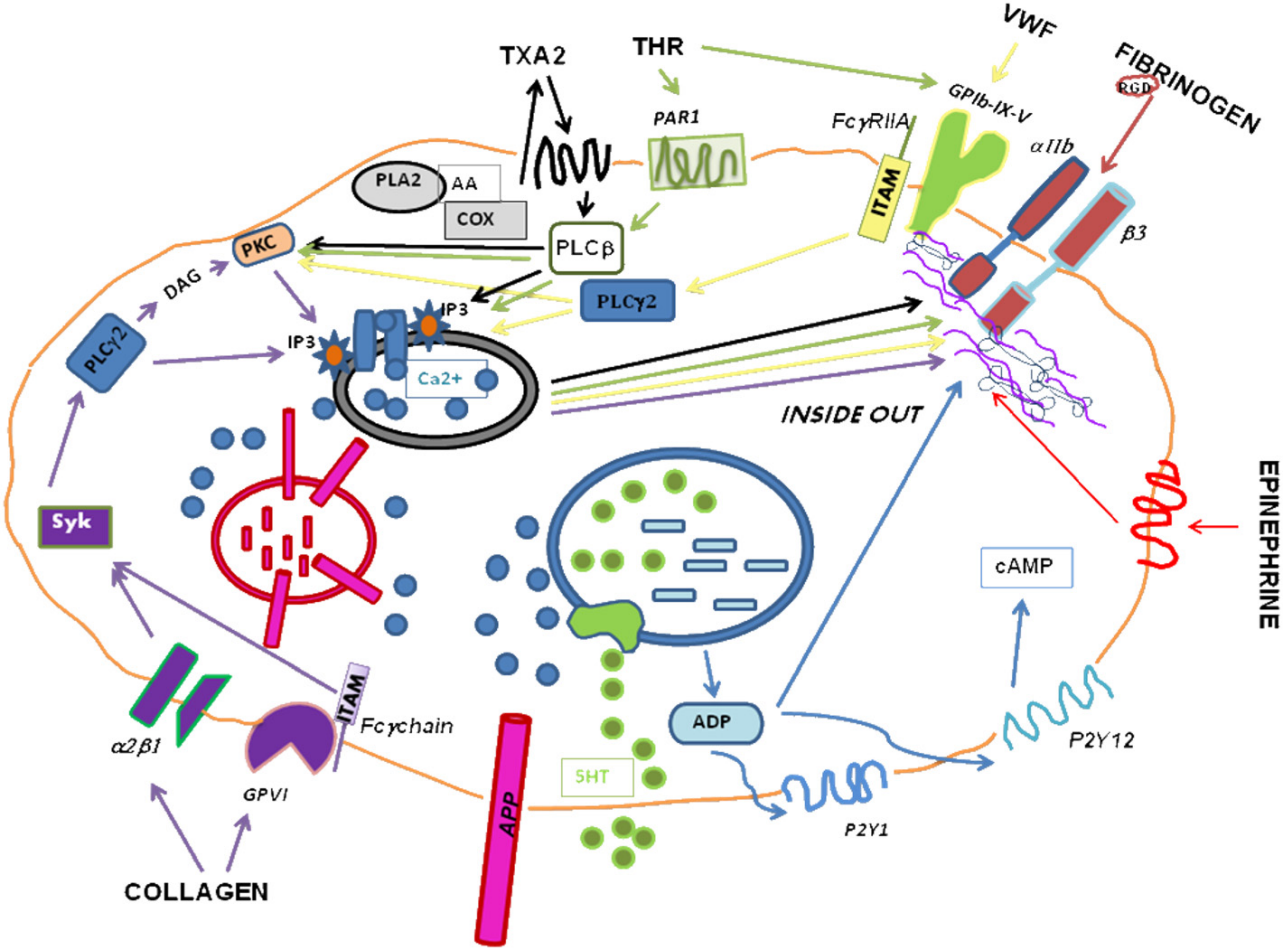
**Principal platelet membrane receptors and signal transduction pathways.** Different receptors are stimulated by various agonists, almost converging in increasing intracellular Ca^2+^ concentration. Platelet activation induces an inside out signaling pathway that active α_IIb_β_3_ integrin. The subsequently link of α_IIb_β_3_ with fibrinogen lead to an outside in signaling pathway that promotes irreversible aggregation. Abbreviations: TXA2, tromboxane A2; THR, thrombin; PAR1, protease-activated receptor-1; VWF, Von Willebrand Factor; RGD, arginine, glycine, aspartic acid; GPIb-IX-V, glycoprotein Ib-IX-V; FcγRIIA, cristallizable fragment γ receptorIIA; α_IIb_β_3_, α_IIb_β_3_ integrin; cAMP, cyclic adenosin monophosphate; P2Y12, P2Y12 receptor; P2Y1, P2Y1 receptor; ADP, adenosin diphosphate; 5HT, 5-hydroxytryptamine; APP, amyloid precursor protein; GPVI, glicoprotein VI; α2β1, α2β1 integrin; Syk, Syk tyrosin kinase; PLCγ2, phospholipase γ2; DAG, diacilglicerol; PKC, protein kinase C; IP3, inositol 3-phosphate, Ca2+, calcium, PLA2, phspholipase A2; AA, arachidonic acid; COX, ciclooxigenase; PLCβ, Phospholipaseβ.

Anucleated blood platelets can be considered a peripheral available model to study those metabolic mechanisms, occurring in the central nervous system and related to AD. Moreover, several intracellular signaling pathways, important for platelet activation involve essential molecules, that have also been described to modulate APP processing [26,27]. The major important platelet agonist, thrombin, actives platelets by binding to membrane receptors PAR1, PAR4, and glycoprotein GPIb-IX-V, which is also able to interact with von Willebrand Factor (vWF). Other important platelets agonists include collagen that binds to integrin α2β1 and the glycoprotein GPVI; ephinephrine, tromboxane A2, and ADP, that bind to specific G protein-coupled receptors. Platelet agonists activate specific signaling pathways, involving different molecules and enzymes, which typically lead to a transient increase of intracellular Ca^2+^ concentration. The final step of platelet activation is the inside-out stimulation of αIIbβ3 integrin activation, which binds fibrinogen and triggers an outside-in signaling pathway that promotes stable and irreversible aggregation (Figure 1).

Platelets also store and release neurotransmitters, such as serotonin, glutamate and dopamine [28,29], and some neuron-related proteins such as N-methyl-d-aspartate, NMDA, receptors.

Therefore, many researchers have focused their attention on platelets as a key peripheral element to understand the pathogenesis of AD.

Animal models are often a valuable tool in basic and biomedical research, and several studies on mice, Drosophila melanogaster, Caenorhabditis elegans and two types of fish, the sea lamprey and the zebrafish [30] have provided essential insights into the molecular mechanism of AD. Transgenic mice are also extremely useful to investigate platelet function, however so far no reports have addressed the platelet biology in the most common murine model for AD, such as the trangenic Tg2576 [31,32] or the PD-APP transgenic mice [33,34].

### The metabolism of amyloid precursor protein in platelets

Human platelets contain high levels of APP, which may contribute to more than 90% of the circulating APP [20]. Platelets show concentrations of APP isoforms equivalent to those found in brain [15], but the expression pattern is different: the isoform APP695, which is the most abundant in neuronal tissue, is nearly undetectable in platelets, where the predominant isoforms are APP770 [16] and APP751 [35].

Platelet APP may represent the major source of Aβ detected in whole blood [36], and recent findings have suggested that platelet APP metabolism might also contribute to the accumulation of Aβ in the brain and its vasculature through the blood brain barrier [37,38].

Intact APP is present on the platelet plasma membrane, and is encoded by platelet mRNA [39]. In fact platelet APP is synthesized by the platelet precursor, the megakaryocyte, in the bone marrow, rather than being the result of platelet uptake of circulating APP [16]. Platelets also express all the required enzymatic activities (α, β and γ-secretases) to produce all the APP metabolites (sAPPα sAPPβ and Aβ), that can be stored into intracellular granules [40]. Proteolytic cleavage of platelet APP may occur both within intracellular organelles of the secretory pathway, and on the platelets’ surface [41,42]. sAPPα and Aβ peptides can be stored in α-granules and released by exocytosis upon platelet activation by thrombin or collagen, which induce Ca^2+^-dependent degranulation [16,20,40-42].

### Role of amyloid precursor protein in platelets

The physiological role of APP and of its metabolites in platelets is not yet well understood. The full-length APP may act as a receptor on the platelet surface, thanks to the cysteine-rich domain, KPI [14,43]; platelet APP has also been proposed to be crucial in the regulation of intracellular Ca^2+^ concentration [44].

Several evidence indicate that platelet APP may play a role also in blood coagulation thanks to KPI domain, inhibiting the activity of the blood coagulation factors IXa, XIa, and Xa [45,46]. Some studies indicate a physiological function for platelet-derived APP in wound repair and in the microenvironmental regulation of the coagulation cascade. In fact, APP possesses growth factor activity [47], and recombinant soluble forms of APP were demonstrated to inhibit platelet aggregation and secretion induced by ADP or adrenaline, indicating that platelet degranulation may result in a negative feedback regulation of platelet activation [48].

### Atypical metabolism of amyloid precursor protein in coated-platelets

Coated-platelets are a recently described subset of platelets that originate upon dual stimulation of platelets with collagen and thrombin which retain more full length APP on their surface than platelets activated with a single agonist [49,50]. Prodan and coworkers reported that there is an alteration of APP metabolism associated with the production of coated-platelets. Moreover, an altered production of coated-platelets in AD patients was also documented [51,52]. This finding is in line with earlier works of Davies and coworkers. However they observed an alteration of coated-platelets formation only in the most severely affected AD patients, while Prodan, observed an increased propensity to form coated-platelets in the mildly affected AD patients, that reverted during the progression of the disease [51-53]. In a more recent study was shown that elevated coated-platelet levels in patients with anamnestic MCI are associated with increased risk for progression to AD [54].

### Release of Aβ peptides by circulating platelets

The main species of Aβ released from activated human platelets is Aβ1-40. This is consistent with the observation that the circulating Aβ forms contributing to vascular amyloid deposits are primary composed by Aβ1-40, while the predominant form in neuronal plaques is Aβ1-42 [55-57]. Regarding the origin of Aβ peptides in blood, two major theories have been proposed: Davies and coworkers [58] suggested that circulating Aβ could derive from the central nervous system through the blood-CSF barrier, to become absorbed onto the surface blood cells, mostly platelets [51] and to a significantly lesser extend lymphocytes [18] and monocytes, which contain only about 5% of the total APP in blood [59]. By contrast, others authors proposed that there is an additional release of Aβ from blood cells and from other non-neuronal cells [60,61].

There is no doubt that Aβ peptides are actively released from platelets [62] and this process is significantly modulated by thrombin [63] and by PGE2 [64]. Smirnov and coworkers identified several forms of Aβ peptide processed and secreted by stimulated and non-stimulated human platelets, including not only the classical Aβ peptides 1–40, 1–42, but also several additional shorter, carboxyl-terminally truncated forms (Aβ1-39, Aβ1-38, Aβ1-37, Aβ1-34), an amino-terminally truncated form (Aβ2-42) and a further not identified form, arbitrarily termed Aβ1-3X, which is probably an oxidized form of Aβ1-40 [62]. All these different forms appear to be released with different kinetics. These observations are in agreement with the findings that a highly conserved pattern of Aβ peptides (Aβ1-37, Aβ1-38, Aβ1-39, Aβ1-40, Aβ1-42 ) was also described in cerebrospinal fluid [65,66].

### Platelet activation by Aβ peptides

Synthetic peptides have been very useful to study the pathophysiological properties of Aβ. Aβ25-3 is a synthetic peptide of 11 aminoacids located in the intermembrane domain of APP [21]. Aβ25-35 cannot be produced through typical APP processing, but is often selected as an alternative model to full-length Aβ because it retains both its physical and biological properties; it aggregates with time, forming fibrils with β-structure [67] and retains the toxicity of the full-length peptide [68,69]; moreover its short length readily allows derivatives to be synthesized and studied [70].

Aβ was able to activate platelets, and to trigger platelet aggregation by stimulating intracellular signaling pathways involving PLCγ_2_ phosphorylation, PKC activation and Ca^2+^ intracellular mobilization. Release of Aβ by activated platelets may represent a mechanism whereby Aβ deposition in the walls of blood vessels leads to angiopathy occuring in aging and AD [71,72].

In vitro experiments showed that low doses of Aβ can potentiate agonist-induced platelet aggregation, and that higher doses are sufficient to directly trigger complete platelet aggregation [73]. These effects were similarly elicited by both Aβ25-35 and Aβ1-40 peptides, and have been linked to specifically signaling pathways [74]. The authors suggested a specific signaling pathway that initiates with the activation of the thrombin receptor PAR1 by Aβ and leads to the subsequent subsequent Ras/Raf, PI3Kinase and Akt cascade activation. Subsequent activation of p38MAPK leads to the stimulation of cPLA_2_, which catalyses the release of arachidonic acid for TXA_2_ synthesis [75]. TxA2 is then able to trigger activation of platelets and the consequent secretion of Aβ, raising the possibility that Aβ activation of platelets may initiate a vicious cycle of platelet activation and Aβ release, which may play a relevant role in the development of cerebral amyloid angiopathy [73].

More recent works established a structure-activity relationship between the polymerization state of Aβ1-40 and its effects on platelet function: fibrillar Aβ1-40 increases ADP stimulated 5-HT serotonin efflux [76]. In the presence of plasma Aβ1-40 fibrils were unable to potentiate platelet aggregation. Perhaps the interactions between plasma lipoproteins and Aβ peptides may represent a protective mechanism that reduces or blocks Aβ toxicity [77]. It also been shown that Aβ1-40 fibrils cause platelets activation supporting platelet adhesion and aggregation via a mechanism that may involve the expression of platelet fibrinogen receptors [74,75].

### Platelets injury and Aβ release

Megakaryocytes are responsible for the production of platelets, by a massive cellular reorganization that leads to the formation of proplatelets. Proplatelets are extruded into the circulation where shear forces trigger their fragmentation, resulting in the release of platelets [76]. It is widely accepted that to produce platelets, megakaryocytes deliberately activate apoptosis [77-80]. Moreover, recent studies have suggested that circulating platelets themselves can undergo apoptosis [81-83].

Activation of apoptotic pathways in platelets can be also stimulated with the proapoptotic agent ionomicyn. This leads to a significant increase in intraplatelet Aβ40, but not Aβ42 [63], leading to the hypothesis that activation of apoptotic pathways in platelets determines an altered processing of APP. Caspases are centrally involved in apoptosis, and some reports indicate the presence of caspase family enzymes in platelets and their activation by extracellular agonists or during prolonged storage [84,85]. By western blot analysis, Casoli and coworkers detected inactive procaspase-3 in resting platelets. Interestingly, in ionomycin-treated platelets the proteolysed and active form of caspase-3 was detected [86], suggesting the initiation of apoptosis that eventually leads to overproduction of Aβ1-40. Thus, Aβ peptide production can be viewed as a consequence of a regular programmed cellular death.

### Do platelets play a role in inflammatory processes in AD?

Besides having a key role in primary hemostasis, platelets play an important role in inflammatory processes. Among the most potent inflammatory signaling molecules secreted by platelets there are chemokines (such as platelet factor 4, PF4, RANTES, and MIP-1α, interleukins (IL-1β, IL-7 and IL-8), prostaglandins and CD40L [87]. RANTES has been identified in platelets α-granules, are released after activation and their secretion from PBMC is increased in AD [88]. MIP-1α is a chemokine found in platelet α-granules too. Its high levels in T-cells and brain microvessels of AD patients suggests that its upregulation could involve also platelets [89]. The platelet endothelial cell adhesion molecules, PECAM-1 and ICAM-1, were shown to be higher in plasma of AD patients versus controls [90]. These proteins are present in platelets and participate in trans-endothelial migration of leukocytes when Aβ peptide acts as an inflammatory stimulus [91,92].

The uncontrolled activation of platelets in AD patients can result in a chronic inflammatory reaction that can mediate endothelial cell stress. This, in turn, may determine further platelet activation creating a vicious circle that causes increased inflammation and release of Aβ. An alternative hypothesis is that the systemic inflammation present in AD determines platelet activation, stabilizing a process that proceeds in a self-amplifying way [89].

### Alteration of platelet sructure and function in ad

**Figure 2:**
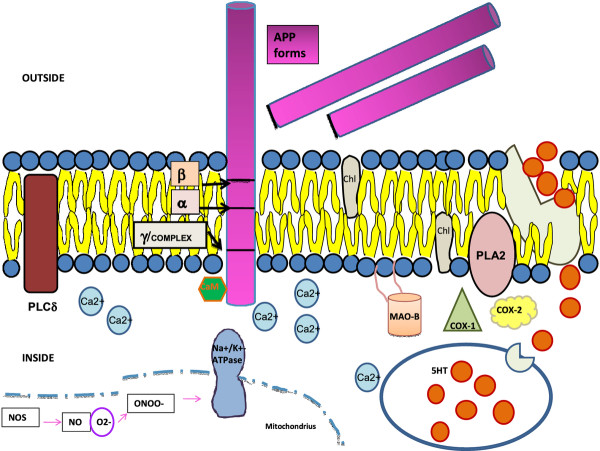
**Abnormalities on AD platelets. Some membrane (secretases, phospholipases), cytosolic (monoamine oxidase, ciclooxigenases) and mithocondrial activities (nitric oxide synthase, sodium potassium ATPase pump) are compromised in AD platelets.** Alterations are evident in the APP processing itself, membrane fluidity and cholesterol levels; in serotonin levels/uptake and intracellular Ca^2+^ levels; in nitric oxide and peroxynitrite production. Abbreviations: PLCδ, phospholipase C δ; β, β-secretase; α, α-secretase; γ-secretase complex; APP forms, amyloid precursor protein forms; CaM, calmodulin; chl, cholesterol; MAO-B, monoamino-oxidase B; PLA2, phosholipase A2; COX-1, ciclooxigenase-1, COX-2, ciclooxigenase-2; 5HT, 5-hydroxytryptamine; NOS, nitric oxide synthase; NO nitric oxide; O2^-^ superoxide anion; ONOO^-^ peroxynitrite; Na+/K + −ATPase, sodium potassium ATPase pump; Ca2+, calcium.

It is now clear that platelet APP processing in AD patients is altered compared to normal control subjects, and may represent a useful peripheral bio-marker for the diagnosis of AD (Figure 2).

### Secretases activities

In contrast to cells of neuronal origin, which predominantly process APP via the β-secretase pathway, platelets, like other non-neuronal cells, favor processing by α-secretase. It is well established that sAPPα concentration in platelets is much higher than Aβ peptides [40].

In fact in addition to cause degranulation and release of stored APP soluble fragments, platelet stimulation promotes primarily the proteolysis of intact APP expressed on the cell surface through α-secretase activity. This process is supported by ADAM metalloproteinases, and causes the release of sAPPα. In co-immunoprecipitation and pull-down experiments a physical association between the intracellular Ca^2+^ sensor calmodulin and APP was documented, revealing a key role of calmodulin in the regulation of non-amyloidogenic processing of APP [93].

In a previous study Smith and collaborators, observed that α-secretase activity did not correlate with age, but it remained constant during the entire lifespan, while β-secretase activity in normal subjects significantly increased with age [94]. Ageing is recognized as being the principal risk factor for AD [95], and it is possible that the pathological changes occurring in AD may initiate very early in life. Moreover the increase of platelet β-secretase activity did not correlate with mini-mental state examination (MMSE) score, indicating that it did not occur as a secondary result of the disease, and may even have preceded the onset of the symptoms [96]. These findings corroborate what already founded for the brains and cerebrospinal fluid of patients affected by AD [97-99]. Other authors reported that platelets from AD patients actually express increased BACE1 activity compared to controls [100,101]. In agreement with the increased β-secretase activity and decreased α-secretase activity, the Aβ levels have been found to be elevated in AD platelets samples [102]. At the present, there are no studies reporting alteration of the γ-secretase activity in AD patients.

### Membrane fluidity and cholesterol levels

Some years ago an alteration of platelet membrane fluidity in AD was reported. This alteration had been ascribed to alteration of the internal membranes rather than to abnormal phospholipids synthesis [103-107].

A slight decrease in platelet membrane cholesterol level in a small group of AD patients was reported [108]. Recent evidence [109] proposed that increased membrane cholesterol results in increased β-secretase activity, that can generate more Aβ1-40. As predicted by other studies [110], increased Aβ1-40 may promote inhibition of HMG-CoA reductase, and reduce de-novo cholesterol biosynthesis. This model hypothesizes a negative feedback system between membrane β-secretase activity and membrane cholesterol level in AD. Therefore, it has been suggested that a perturbation of a possible physiological homeostatic link between membrane cholesterol level and membrane β-secretase activity may occur in AD. Treatment with statin may restore this link [109].

### Phospholipases

A key enzyme in platelets signal transduction is the phosphoinositide-specific phospholipase C, PLC. Its involvement in AD dates back some years ago when Shimohama and coworker demonstrated that the PLCδ-1 isoform abnormally accumulates in the brain in AD [111]. Subsequently it was demonstrated that PLC activity was significantly lower in the AD platelets than in controls, suggesting an aberrant phosphoinositides metabolism in non-neuronal tissues [112]. Moreover the activity of PLCδ1 isozyme is reduced in AD patient homozygous for apoE genotype carrying the *ε*3 allele, but was normal in patients with the *ε*4 allele [113].

Phospholipase A_2_, PLA_2_, plays an essential role in the metabolism of membrane phospholipids [114], but its activation can also stimulates the secretion of APP [115], and, on the other hand amyloid peptides are able to activate PLA_2_ in vitro [116]. PLA_2_ activity was found to be increased in platelets from individuals with AD [117]. These data conflict with those of other studies in which PLA_2_ activity in human platelets [118] as well as in human brain AD [119,120] was found to be decreased in AD samples.

### Serotonin levels and uptake

Several studies have reported abnormalities in the cellular content of serotonin in AD, as well as alterations of its uptake in different regions of the brains of AD patients [121,122]. Studies performed on AD platelets reported confused results concerning the uptake of serotonin. While some works failed to detect any alteration in the level of serotonin in AD patients compared to controls [123], others documented a reduced uptake of serotonin into AD platelets [124,125].

In accord to the diminished platelet serotonin concentration and increased plasma serotonin levels in patients with AD, Sevush and coworkers, observed that unstimulated platelets of AD patients exhibit greater activation of than those of controls [126]. A recent work shows a persistently enhancement of platelets activation of AD patients, which may be related, on increased lipid peroxidation associated with inadequate levels of Vitamin E [127].

A straight correlation between the uptake of 5-hydroxytryptamine, 5-HT and cognitive state of the AD patients was also reported. A significantly lower concentrations of serotonin in platelets from AD patients was peculiar of those subjects in the late phase of AD. Hence, the decreased platelet serotonin concentration observed in the late phase of AD might be related to a reduced serotonin uptake [128].

### Monoamine oxidase activity

Few studies performed on platelet MAO-B activity in AD yielded inconsistent but intresting results: Adolfsson and coworkers report increased MAO-B activity in platelets and brain of AD [129], while Ahlskog did not find any alteration of MAO-B activity in AD versus controls [130]. More recent studies indicated that MAO-B activity might be used as a biomarker for the presence of psychotic features in AD [131], and for the early or late onset AD [132]. In the above cited work by Muck-Seler and coworkers, the MAO-B activity was in line with serotonin concentration: significantly lower in patients in the late phase of AD compared to other phases of AD or healthy controls. Authors have found a positive correlation between MMSE scores and platelet MAO-B activity in AD, thus indicating that more severe AD symptoms are associated to lower MAO-B activity [128].

### Cyclooxygenase

Current data suggest that protein kinase C, along with PI3K activity and Ca^2+^, is crucially involved in APP cleavage and secretion in human platelets, while COX is a minor component of APP secretion pathway. By contrast, Aβ release is totally independent of both PKC and COX activity, meanwhile Ca^2+^ plays an important role also in the release of Aβ [54].

Other evidence linked to the action of COX enzymes in brains, suggest a determinant role of COX as inflammatory marker in peripheral cells of AD, as platelets.

Activated platelets express almost exclusively, COX-1. COX-2 isozyme is normally undetectable in most tissue, but can be rapidly induced by proinflammatory or mitogenic stimuli. Elevated concentrations of circulating cytokines could upregulate COX-2 in megakaryocytes with a subsequent increase of it content in platelets. Bermejo and coworkers, reported an increased platelets levels of COX-2 in AD and MCI patients compared to elderly controls, indicating that platelet inflammatory pathways are activated, and that this could be considered an early event in AD development [133].

### Nitric oxide synthase and free radicals generation

Oxidative stress has been widely implicated both in ageing and in pathogenesis of several neurodegenerative disorders, including AD. Some studies showed an increase of nitric oxide, NO and peroxynitrite anion, ONOO^-^, production exclusively in platelets obtained from AD patients, that was associated to a decreased Na^+^/K^+^-ATPase activity in AD patients platelet membrane [134]. In a vertical study, nitric oxide syntase, NOS, activity, was measured in platelets of young controls, aged controls and AD patients, and a significant increase was seen when AD were compared with aged controls and, more significant, when compared with young controls [135]. In a study performed on mild and moderate AD patients, platelet aggregation was found to be potentiated, and this was paralleled by a decline of endothelial, constitutive NOS activity, causing a reduction of the NO concentration in platelets. This reduction was proposed to be responsible for the increased aggregation in AD patients, since NO can inhibit platelet aggregation [136]. This model, however, is in contrast with the well documented notion that NO concentration and NOS activity is higher in AD than controls. Therefore, additional works are necessary to determine the role of NO system in AD platelets.

### Alteration of APP processing

The alteration of APP processing in platelets from AD patients was investigated at least by three different groups, who measured the ratio between the different types (or isoforms) of APP detectable in platelets on the basis of a different apparent molecular mass on SDS-PAGE.

Rosenberg and coworkers determined the ratio of the 120–130 KDa APP isoform to the 110 KDa APP isoform, and showed that APP processing in AD platelets is compromised compared to that of control subjects [101,137,138]. The authors propose that this difference may reflect chronic platelet activation in patients with AD. They also determined a direct correlation between the cognitive decline by MMSE scores in AD patients during three years of follow-up and APP isoform ratio reduction.

Liu and coworkers revealed that AD patients whose MMSE scores declined in one year, had a greater reduction in platelet APP isoforms ratio than patients whose MMSE scores did not decline [139].

An altered ratio among the different APP isoforms detectable in platelets from AD patients was confirmed by many other studies [59,140,141], as it was the demonstration of a positive correlation between APP ratios and the progression of clinical symptoms, suggesting that this peripheral parameter may be a marker of progression of the disease.

Subsequently studies reported that platelets of patients carrying the mutation Met293Val in PS2 protein did not show altered expression of APP isoforms ratio pattern conversely to what reported for sporadic AD patients [142].

It was demonstrated an association between early stages of AD, or of Mild Cognitive Impairment, MCI, with a reduction in platelet APP isoforms ratio, and suggested that, since alteration of APP processing may be an early event in AD, the characterization of APP ratio in platelet could have a great diagnostic power [8,143-145]. In conclusion, platelet APP isoforms ratio may be a very important AD biomarker, as its evaluation is reliable and simple test to be performed.

## Conclusion

Initially identified as an exclusively brain tissue disorder, in the last decades, AD, was revaluated as a more intriguing disorder involving many other peripheral tissues and molecules in the organism. In fact, it is now well established that biochemical alterations in AD patients do not occur only in the brain, but even in blood vessels and blood cells. Nowday researchers have recognized that platelets are the principal components of human blood to be affected not only in the onset but even in the progression of AD. Platelets seem to mirror what happens in nervous tissue during the evolution of AD, and therefore represent the cellular type where to identified the early events in the onset of the disorder. Differently from neurons, platelets can be easily accessible and constitute a valid cellular tool to study the pathogenesis of AD. In this review we have summarized the limited and often discordant results reported by different authors in recent years. Overall all these studies, both in vivo and in vitro, are aimed to understand in which way affected proteins, enzymes, signal transduction pathways, inflammatory processes, spontaneous activation are important in platelets to better define the molecular pathogenesis of AD. Platelets are primary the principal authors of hemostasis in the organism, but they also play a key role in Alzheimer Disease still remaining a potential marker to understand the diagnosis of the disorder.

## Competing interests

The authors declare that they have no competing interests.

## Authors' contributions

SC wrote the manuscript. MT and GR edited the manuscript. All authors read and approved the final manuscript.
